# Electrochemiluminescence Sensor Based on Electrospun Three-Dimensional Carbon Nanofibers for the Detection of Difenidol Hydrochloride

**DOI:** 10.3390/s19153315

**Published:** 2019-07-28

**Authors:** Hao Cheng, Zhengyuan Zhou, Yanqing Li, Wenyi Huang, Jun Feng, Tingfan Tang, Lijun Li

**Affiliations:** 1 Guangxi Key Laboratory of Green Processing of Sugar Resources, College of Biological and Chemical Engineering, Guangxi University of Science and Technology, Liuzhou 545006, Guangxi, China; 2 Province and Ministry Co-Sponsored Collaborative Innovation Center of Sugarcane and Sugar Industry, Nanning 530004, Guangxi, China

**Keywords:** carbon nanofiber, difenidol hydrochloride, electrochemiluminescence, Ru(bpy)_3_^2+^

## Abstract

The detection of difenidol hydrochloride, which is a drug that is widely used for treating the nausea and vomiting symptoms caused by certain diseases, has been increasingly involved in cases of suicide via overdosing and of drug poisoning in children. A novel electrochemiluminescence (ECL) sensor for the simple and effective detection of difenidol hydrochloride was fabricated by modifying a glassy carbon electrode with three-dimensional carbon nanofibers (3D-CNFs). The 3D-CNFs were synthesized by electrospinning a mixture of montmorillonite (MMT) and polyacrylonitrile, carbonizing the electrospun product, and etching it with hydrofluoric acid. The form and structure of the 3D-CNFs was analyzed via scanning electron microscopy, X-ray photoelectron spectroscopy, and Raman microspectroscopy. According to the experimental results obtained using the modified electrodes, a good linear relationship was found between peak intensity and difenidol concentration (y = 868.14x − 61.04, R^2^ = 0.999), with a relatively low detection limit (8.64 × 10^−10^ mol·L^−1^ (S/N = 3)). In addition, our approach exhibited good recovery values ranging from 98.99% to 102.28%. The proposed novel ECL sensor has wide application prospects for the detection of difenidol hydrochloride.

## 1. Introduction

Difenidol hydrochloride (1,1-diphenyl-4-piperidino-1-butanol hydrochloride) is used to relieve or prevent dizziness, nausea, and vomiting [1]. Since its first approval in the United States in 1967 [2], this drug has been widely used in China and has contributed to the treatment of labyrinthitis, vestibular neuronitis, and Meniere’s disease [3]. It is believed to diminish vestibular stimulation and depress nausea and vomiting at the medullary chemoreceptor trigger zone [4]. Cases of drug poisoning in children and of adults taking the drug to commit suicide have been recently reported. Thus, there is an increasingly urgent need to develop methods for the detection of difenidol hydrochloride. High-performance liquid chromatography (HPLC) [5,6], chromatography-mass spectrometry (LC-MS) [7], post-chemiluminescence [8], anionic surfactant titration [9], and microfluidic chips [10] are common approaches for detecting difenidol hydrochloride. However, these techniques are time-consuming and require complex and expensive instruments, as well as specialized personnel to operate them. In addition, lack of sensitivity is another concern. Therefore, it is necessary to find a more effective, sensitive, and simpler approach for detecting difenidol hydrochloride.

Electrochemiluminescence (ECL), which is a kind of luminescence produced during electrochemical redox reactions, combines the advantages of chemiluminescence and electrochemistry [11]. It has a wide range of applications [12] owing to the simple equipment required and its high sensitivity [13], rapid detection times, and ease of operation [14,15,16]. ECL is a field of growing interest and has been widely applied in various areas, including biology [17,18], environmental science [19], and pharmacology [20,21]. Tris(2,2′-bipyridyl)ruthenium(II) (Ru(bpy)_3_^2+^) is one of the most widely used chemiluminescent reagents in ECL systems because of its stability and efficiency [22].

It is possible to chemically modify electrodes to change the microstructure of their surface, and in turn, enhance the sensitivity and selectivity of ECL-based sensing devices. The enhancement of ECL systems through electrode surface modification is expected to extend the research scope of ECL and facilitate its application for drug detection. Electrospun carbon nanofibers (CNFs) have become a promising candidate among potential materials for electrode modification owing to their high surface-area-to-volume ratio [23,24,25], high strength, excellent electrical conductivity, and high number of edge plane defects on the outer wall, which facilitates electron transfer [26,27,28].

It has been established in previous studies that ECL and capillary electrophoresis-electrochemiluminescence (CE-ECL) can be used to detect difenidol hydrochloride [29,30]. The working electrodes respectively used for these two approaches are multi-walled carbon nanotube-modified glassy carbon electrodes (MWCNTs/GCEs) and platinum (Pt) electrodes modified with gold nanostructures. However, carbon nanofiber-modified glassy carbon electrodes represent a new kind of sensor that has not been previously reported.

**Scheme 1:**
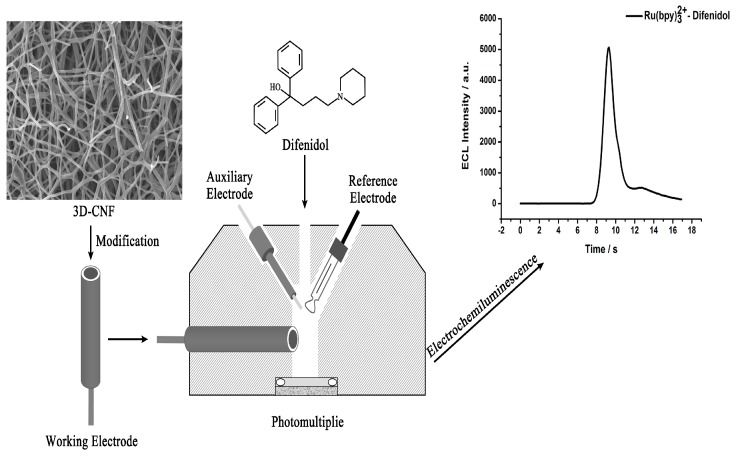
Preparation of the three-dimensional networks of carbon nanofibers (3D-CNF/GCE) and the detection process for difenidol.

In this study, montmorillonite (MMT) was chosen as a proppant and mixed into an electrospinning precursor solution. After electrospinning, carbonization, and etching with hydrofluoric acid (HF), three-dimensional (3D) networks of carbon nanofibers (3D-CNFs) were obtained. These kinds of 3D networks contain numerous active sites, which accelerate electron transfer. After modifying the surface of a glassy carbon electrode (GCE) using these 3D networks, an ECL sensor capable of difenidol hydrochloride detection was obtained. Scheme 1 shows the fabrication process of the glassy carbon electrode modified by three-dimensional networks of carbon nanofibers (3D-CNF/GCE) and the detection procedure for difenidol.

## 2. Experiments

### 2.1. Reagents

A standard stock solution with a concentration of 1.5 × 10^−3^ mol/L of difenidol hydrochloride was prepared by dissolving difenidol hydrochloride (National Institutes for Food and Drug Control (NIFDC), Beijing, China) into ultrapure water and was stored in a refrigerator. A stock solution of Ru(bpy)_3_^2+^ was prepared by dissolving Ru(bpy)_3_Cl_2_·6H_2_O in ultrapure water. Polyacrylonitrile (PAN; molecular weight: 85,000 g/mol, Sigma-Aldrich Co., Ltd. Shanghai, China) was used as the carbon source for the nanofibers. *N*,*N*-dimethylformamide (DMF), acetone, and Nafion solutions (5 wt.%) were purchased from Sigma-Aldrich (Shanghai, China). All the chemicals used in the experiments were of analytical grade, and ultrapure water was used. A 0.1-M borate buffer solution (pH = 7.5) was prepared by mixing a 0.1-M H_3_BO_3_ solution and a Na_2_B_4_O_7_·10H_2_O solution.

### 2.2. Instruments

Field-emission scanning electron microscope (FE-SEM; CarlZeiss, Germany) was used to observe the external morphology of the synthesized materials. An X-ray photoelectron spectroscope (XPS, Kratos Analytical Ltd., UK) was used to determine the elemental composition of the materials’ surface layer. A laser confocal Raman microspectroscope (Raman, XploRA PLUS, Horiba) was used to measure the Raman spectra of the materials.

### 2.3. Synthesis of CNFs

The precursor solution for the electrospinning process was prepared as follows: 1.6 g of PAN powder and 15-wt.% MMT were dissolved in 10 mL of DMF via magnetic stirring for approximately 12 h at room temperature. As a result, a solution of PAN/MMT composites was obtained. The electrospinning process, which results in nanofiber formation, was carried out by applying a high voltage of 15 kV. After drying in a vacuum for 12 h, the composite nanofibers were calcinated in N_2_ at 800 °C at a heating rate of 2 °C/min. They were then left to naturally cool down to room temperature. Subsequently, the composite nanofibers were etched with 10% HF and rinsed with deionized water until a neutral pH was reached. After drying at 80 °C overnight, the final product was obtained.

### 2.4. Fabrication of CNFs/GCEs

Prior to the fabrication of the final electrode, the GCE was polished until it became a mirror-like surface using fine emery papers and 0.3- and 0.05-µm alumina slurry. Afterwards, the GCE was cleaned ultrasonically using deionized water and ethanol alternatively, and then dried in a N_2_ atmosphere. Subsequently, 1.5 mg of CNFs were dispersed via sonication in isopropanol-H_2_O (1:4, *v/v*) to produce a homogeneous and stable suspension. Then, 5 µL of the prepared suspension were pipetted onto to the polished surface of the GCE, followed by drying at room temperature. Through this process, the modified GCE was obtained.

### 2.5. Electrochemical and Electrochemiluminescence Measurements

In this study, all electrochemical (EC) and ECL measurements were performed using a MPI-E electrogenerated chemiluminescence analyzer (Xi’an Remex (Ruimai) Analysis Instruments Co., Ltd., Xi’an, China) with a three-electrode cell. The working electrode was a GCE (Φ = 3 mm), the counter electrode was a Pt wire, and the reference electrode was made of Ag/AgCl. Cyclic voltammetry (CV) measurements were carried out to determine the EC and ECL properties of difenidol hydrochloride. The sample was put in a borate buffer solution (0.1 M, pH = 7.5) under optimal conditions; a high negative voltage of −800 V was applied to the photomultiplier, the scan rate was 100 mV/s, and the concentration of Ru(bpy)_3_^2+^ was 4 × 10^−4^ M.

## 3. Results and Discussion

### 3.1. Drug Substance Characterization

**Figure 1:**
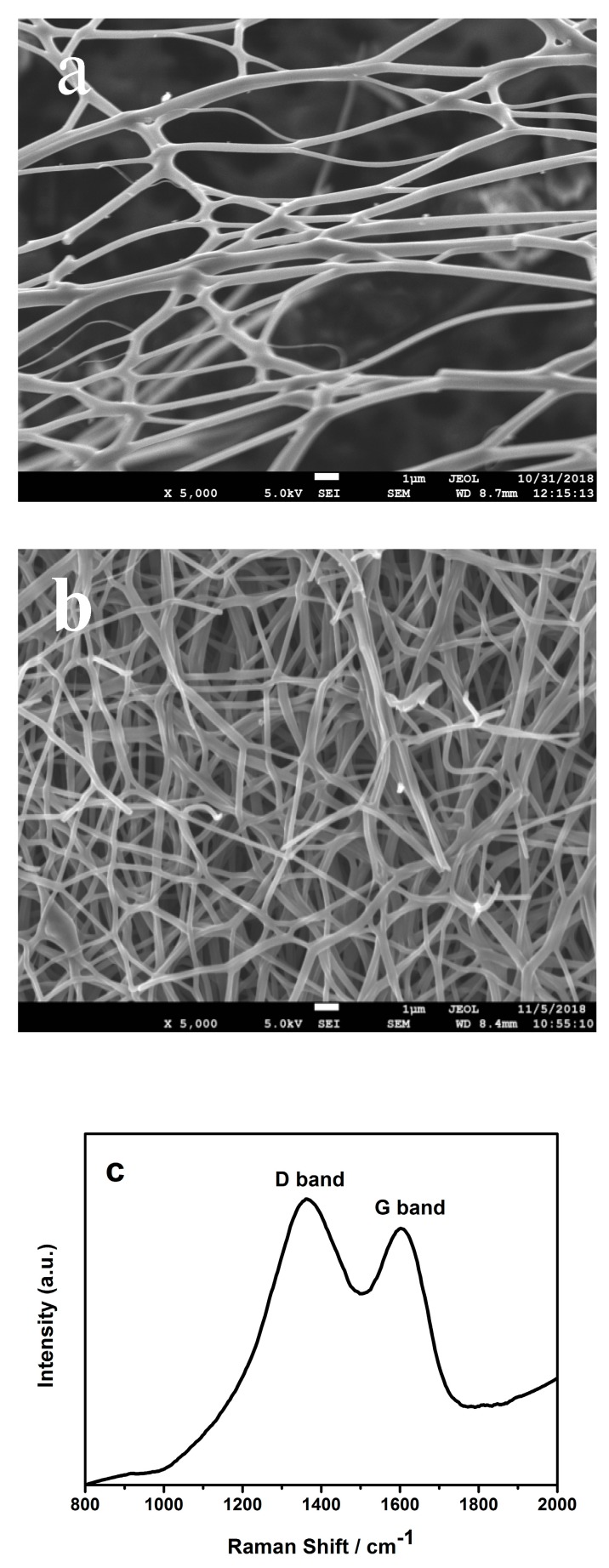
SEM images of carbon nanofibers (CNFs) (**a**) and 3D-CNFs (**b**) and Raman spectra of 3D-CNFs (**c**).

As shown in Figure 1a,b, the morphological differences between CNFs and 3D-CNFs can be observed via scanning electron microscopy. The CNFs exhibited a planar reticular structure before the addition of MMT. After MMT was added, the substances formed a well-defined and interconnected 3D porous network. Such interconnected networks can facilitate the detection of difenidol hydrochloride because they contain a high number of active sites, which accelerates diffusion [31].

**Figure 2:**
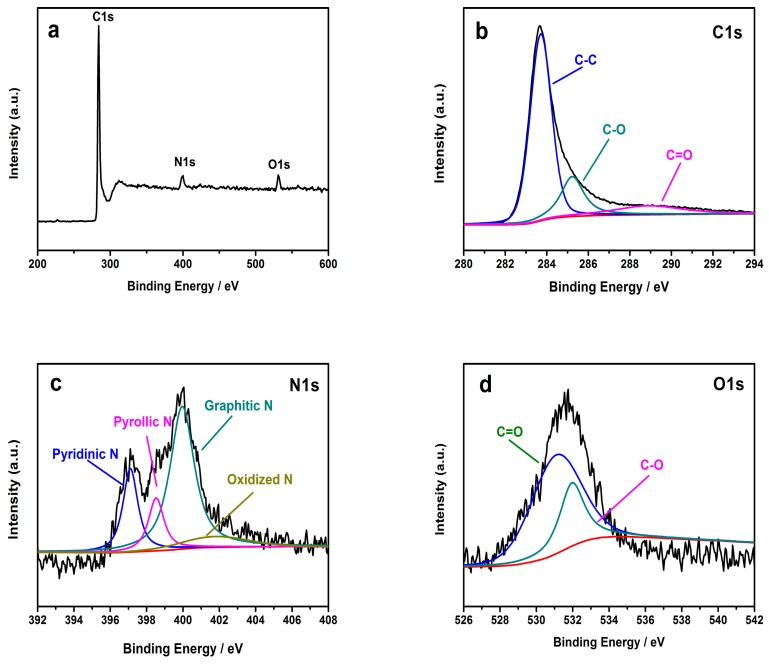
XPS spectra of 3D-CNFs (**a**) and high-resolution XPS spectra for C1s (**b**), N1s (**c**), and O1s (**d**).

The elemental composition of the sample surface was determined via X-ray photoelectron spectroscopy. The results are presented in Figure 2. According to the XPS spectrum of the sample (Figure 2a), the peaks that appeared at 284.8 eV, 399 eV, and 532 eV can be assigned to C1s (89.51 at.%), N1s (5.43 at.%), and O1s (3.24 at.%), respectively [32]. The deconvoluted high-resolution C1s spectrum (Figure 2b) revealed the presence of three peaks at 284.5 eV, 285.8 eV, and 289 eV, which were attributed to C=C, C–C, and C=O, respectively [33,34]. The deconvoluted high-resolution N1s spectrum (Figure 2c) revealed four main peaks at 398.5 eV, 399.9 eV, 401.5 eV, and 402 eV, which were attributed to different types of nitrogen: pyridine, pyrrolic, graphitic, and oxidized nitrogen, respectively [35,36]. As shown in Figure 2d, O1s peaks of high intensity were observed at 531.8 eV and 533 eV, which were attributed to C=O and C–O, respectively [37].

Raman spectroscopy was performed to obtain detailed information about the chemical structure of the sample. The disorder-induced D band (graphite) and the order-induced G band (graphite) were observed in the measured Raman spectrum. As shown in Figure 1c, the D band and the G band appeared at 1380 and 1600 cm^−1^, respectively [38,39]. The relative intensity of the D and G bands (I_D_/I_G_) was 1.05, indicating a low degree of graphitization and a high degree of disorder in the sample [40,41].

### 3.2. EC and ECL Behaviors of Difenidol Hydrochloride

#### 3.2.1. ECL Behavior of Difenidol Hydrochloride on 3D-CNFs-Modified GCEs

**Figure 3:**
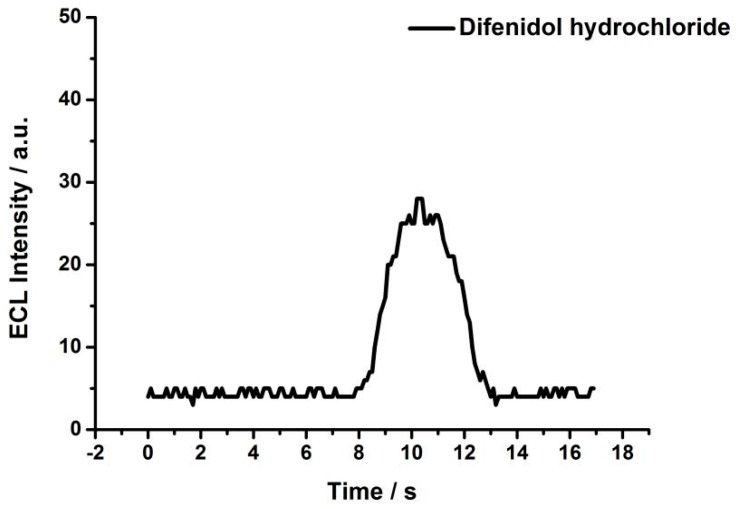
ECL intensity–time curve of difenidol hydrochloride in a solution with 3 × 10^−5^ mol·L^−1^ of difenidol and 0.1 mol·L^−1^ of BBS (pH 7.5).

As shown in Figure 3, when trying to detect difenidol hydrochloride using the 3D-CNFs/GCE without Ru(bpy)_3_^2+^, no significant electrochemical response was observed. This proves that difenidol hydrochloride has poor ECL properties.

#### 3.2.2. EC and ECL Behaviors of Ru(bpy)_3_^2+^ and Ru(bpy)_3_^2+^-Difenidol Hydrochloride on 3D-CNFs-Modified GCEs

**Figure 4:**
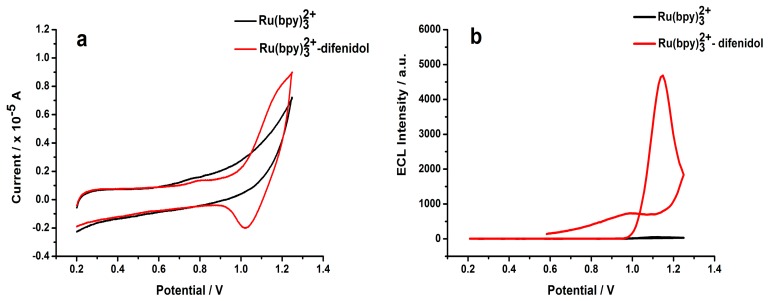

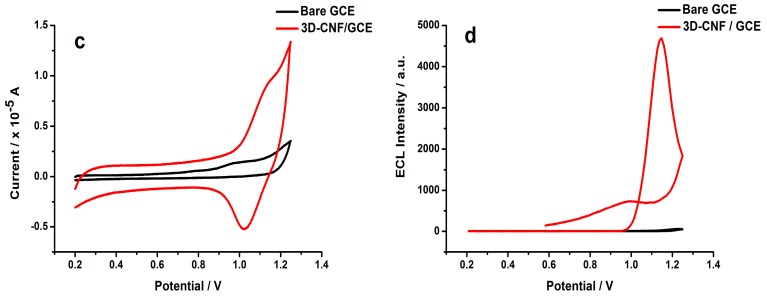
EC (**a**) and ECL (**b**) curves of Ru(bpy)_3_^2+^ and Ru(bpy)_3_^2+^-difenidol hydrochloride in a 0.1-mol·L^−1^ borate buffer solution (pH 7.5), and EC (**c**) and ECL (**d**) curves of Ru(bpy)_3_^2+^-difenidol hydrochloride in a 0.1-mol·L^−1^ BBS solution (pH 7.5) on a bare GCE and a 3D-CNF GCE, respectively.

As shown in Figure 4a, the oxidation peak was identified at +1.0 V and increased when difenidol hydrochloride was added to the electrolyte solution. This was due to the synergetic effect of difenidol hydrochloride on the oxidation–reduction reactions of Ru(bpy)_3_^2+^ [42], by which the tertiary amino of difenidol was oxidized into radical cations.

ECL was generated via annihilation. The ECL response of Ru(bpy)_3_^2+^ was measured and found to be weak; the results are shown in Figure 4b. The corresponding ECL mechanism is shown in Equations (1)–(4). In contrast, when difenidol was added, ECL intensity was greatly enhanced. This occurred because difenidol not only transfers electrons to the surface of the electrodes, but also produces highly reducible intermediates with great energy, such as free radicals. This mechanism can be expressed as Equations (5)–(11) [43,44,45,46].

Ru(bpy)_3_^2+^ − e^−^ → Ru(bpy)_3_^3+^ (1)

Ru(bpy)_3_^2+^ + e^−^ →Ru(bpy)_3_^+^ (2)

Ru(bpy)_3_^3+^ + Ru(bpy)_3_^+^ → Ru(bpy)_3_^2+^ + Ru(bpy)_3_^2+*^ (3)

Ru(bpy)_3_^2+*^ → Ru(bpy)_3_^2+^ + *hv* (4)

C_20_H_24_OCH_3_N − e^−^ → C_20_H_24_OCH_3_N^+^ (5)

C_20_H_24_OCH_3_N^+^ → C_20_H_24_ONC^*^H_2_ + H^+^ (6)

Ru(bpy)_3_^2+^ − e^−^ → Ru(bpy)_3_^3+^ (7)

C_20_H_24_ONC^*^H_2_ + Ru(bpy)_3_^3+^ → Ru(bpy)_3_^2+*^ + C_20_H_24_ON^+^ = CH_2_ (8)

C_20_H_24_ONC^*^H_2_ + Ru(bpy)_3_^2+^ → Ru(bpy)_3_^+^ (9)

Ru(bpy)_3_^3+^ + Ru(bpy)_3_^+^ → Ru(bpy)_3_^2+*^ (10)

Ru(bpy)_3_^2+*^ → Ru(bpy)_3_^2+^ + *hv* (11)

#### 3.2.3. EC and ECL Behaviors of Ru(bpy)_3_^2+^-Difenidol Hydrochloride

Figure 4c,d shows the EC and ECL curves of Ru(bpy)_3_^2+^-difenidol on bare GCEs and 3D/CNF GCEs, respectively. It can be observed that both the chemical current and ECL intensity of Ru(bpy)_3_^2+^-difenidol on the modified electrodes were higher than that on the bare electrodes. Moreover, the ECL intensity of Ru(bpy)_3_^2+^-difenidol on the modified electrodes was approximately 60 times higher than that on the bare electrodes. This is because the 3D-CNFs have a large surface area and many edge plane defects on their outer wall, which enhances the diffusion of difenidol and provides an efficient pathway for electron transfers. Consequently, the charge transfer rate increases.

#### 3.2.4. Diffusion Coefficient of Difenidol on Different Electrodes

To prove that 3D-CNFs can accelerate the diffusion of difenidol, and thereby contribute to enhancing the resulting ECL signal, the diffusion of difenidol on different electrodes was studied via chronoamperometry. The diffusion coefficients of difenidol on the different electrodes were calculated according to the Cottrell equation. As shown in Table 1, the diffusion coefficient of difenidol on the 3D-CNF/GCE was higher than that on the bare GCE, which is consistent with the fact that the ECL intensity of difenidol on the 3D-CNF/GCEs was greater than that on the bare electrodes. This indicates that 3D-CNFs can enhance the diffusion of difenidol.

### 3.3. Optimization of Detection Conditions

After preparing various sensors, we optimized several influencing factors to obtain the best sensitivity for the detection of difenidol hydrochloride. These factors included buffer type and pH value, the volume of 3D-CNFs used for surface modification, the concentration of Ru(bpy)_3_^2+^, the scan rate, and the high negative voltage applied.

#### 3.3.1. Effects of 3D-CNFs Modification

**Figure 5:**
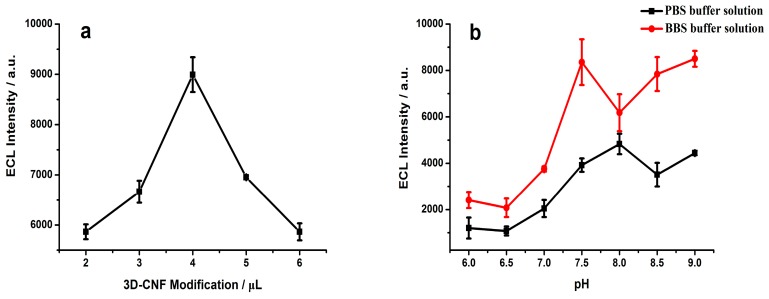
Optimization of detection conditions: (**a**) volume of 3D-CNFs; (**b**) buffer type and pH value. A 0.1-M BBS solution was used (pH = 7.5; with a concentration of 3 × 10^−5^ mol·L^−1^ of difenidol hydrochloride). Error bar: standard deviation for *n* = 3. PBS:phosphate buffer solution; BBS: borate buffer solution.

As shown in Figure 5a, the light signal increased with the volume of 3D-CNFs within the range of 2 to 4 µL. This could be ascribed to the resulting larger surface area and higher number of active sites. For volumes higher than 4 µL, the light signal begins to decrease as the volume increases. This is because excessively thick modification hinders electron transport [47]. Thus, 4 µL was chosen as the optimal volume of 3D-CNFs for surface modification.

#### 3.3.2. Effects of Buffer Type and pH Value

The buffer type and pH value used will greatly determine the stability and signal strength in ECL-based detection systems [48]. Thus, selecting the best buffer solution is vital for the detection of difenidol. The effects of using a phosphate buffer solution (PBS) and a borate buffer solution (BBS) were analyzed. In these experiments, each buffer was designed to contain 3 × 10^−5^ mol·L^−1^ of difenidol hydrochloride. Figure 5b shows the comparative ECL intensities obtained, where it can be seen that BBS exhibited the best performance. The maximum ECL intensity was achieved at a pH of 7.5, because the use of a high pH value helped in the deprotonation of difenidol, and thus enhanced the luminescence signal [42]. Therefore, in subsequent experiments, we adopted BBS as the buffer solution and used it at an optimal pH of 7.5.

#### 3.3.3. Effects of the Concentration of Ru(bpy)_3_^2+^

We hypothesized that the concentration of the luminescent reagent would also affect the ECL intensity of Ru(bpy)_3_^2+^-difenidol. According the experimental data obtained, ECL intensity reached its maximum value when the concentration of Ru(bpy)_3_^2+^ was 4 × 10^−4^ mol·L^−1^, and then fell off as the concentration of Ru(bpy)_3_^2+^ continued to increase. The other influencing factors were controlled; difenidol hydrochloride was used at a concentration of 3 × 10^−5^ mol·L^−1^ in BBS at a pH of 7.5. Therefore, our initial hypothesis was verified. Considering the resulting signal-to-noise ratio (SNR), an optimal concentration of Ru(bpy)_3_^2+^ of 4 × 10^−4^ mol·L^−1^ was chosen.

#### 3.3.4. Selection of Instrument Parameters

To increase the service life of the photomultiplier and ensure the stability and sensitivity of the detection system, the effects of varying the high negative voltage applied and the scanning rate were studied. The other influencing factors were controlled; the pH of the BBS was maintained at 7.5 and difenidol hydrochloride was used at a concentration of 3 × 10^−5^ mol·L^−1^. We found that when the negative high voltage applied was −800 V and the scanning rate was 0.1 V·s^−1^, the measured signal was stable but with a lower SNR. Because the use of a higher scan rate would negatively affect the instrument [12], the aforementioned values were selected as the optimal instrument parameters.

### 3.4. Analytical Performance of the 3D-CNFs/GCEs

**Figure 6:**
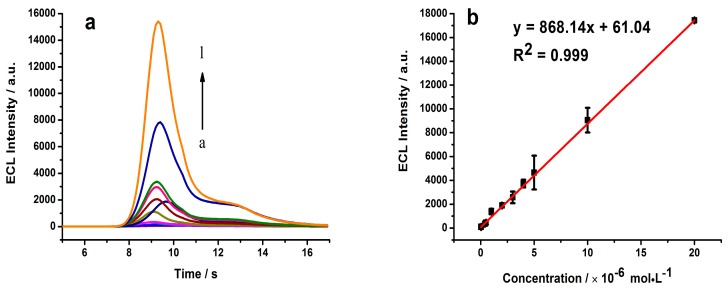
ECL responses of the 3D-CNFs/GCE in a 0.1-M borate buffer solution (**a**) for different concentrations of difenidol hydrochloride ranging from 3 × 10^−8^ to 2 × 10^−5^ mol·L^−1^; (**b**) calibration curves of the ECL responses for different difenidol hydrochloride concentrations. Error bar: standard deviation for n = 3.

Using the optimal conditions stated above, the ECL intensities for different concentrations of difenidol hydrochloride on the 3D-CNFs-modified GCEs were studied using an MPI-E electrogenerated chemiluminescence analyzer. Figure 6a shows that the peak current increased gradually as the concentration of difenidol hydrochloride increased, and there was a good linear relationship between peak intensity and the concentration of difenidol hydrochloride within the range of 3 × 10^−8^ to 2 × 10^−5^ mol·L^−1^. The correlation of these two variables followed y = 868.86x + 61.04, R^2^ = 0.999 (Figure 6b), with a detection limit (LOD) of 8.64 × 10^−10^mol·L^−1^ (S/N = 3). Table 2 shows that the proposed ECL sensor based on a 3D-CNF-modified GCE had a higher sensitivity and stronger linear relationship for the detection of difenidol hydrochloride than traditional methods. Additionally, it also had a lower LOD than other approaches.

### 3.5. Selectivity of the Sensor

An interference study was carried out under the aforementioned optimal conditions. We aimed to analyze the selectivity of the ECL sensor for detecting 1.0 × 10^−^^5^ mol·L^−^^1^ of difenidol hydrochloride. We found that concentrations of Na^+^, K^+^, Cl^−^, and NO_3_^−^ 500 times higher, of glucose, sucrose, and fructose 25 times higher, and of ascorbic acid 5 times higher did not interfere with the detection of difenidol hydrochloride.

### 3.6. Reproducibility and Repeatability of the Modified Electrodes

Five different 3D-CNFs-modified electrodes were prepared to detect difenidol hydrochloride. The results indicated good reproducibility with a relative standard deviation (RSD) of 3.40%. Similarly, the same modified electrode was used repeatedly ten times and its RSD was 3.75%. In other words, 3D-CNF/GCEs have the advantages of both good repeatability and reproducibility.

### 3.7. Analysis of a Real Sample

A solution with a concentration of 1.5 × 10^−6^ mol·L^−1^ of difenidol hydrochloride was obtained by dissolving five crushed tablets of difenidol hydrochloride in deionized water. Appropriate parts of the solution were taken as samples, which were accurately weighed using an analytical balance. Subsequently, the 3D-CNFs/GCE sensor was used for the detection of difenidol hydrochloride in the sample solutions under the optimal conditions. Additional experiments were also carried out as standards. Table 3 shows that the recoveries ranged from 98.99% to 102.28%, demonstrating the good performance of the proposed sensors in practical applications.

## 4. Conclusions

In this study, an ECL sensor was produced by modifying the surface of a glassy carbon electrode with 3D-CNFs. The proposed sensor was successfully used to detect difenidol hydrochloride. Owing to the advantages of using three-dimensional carbon nanofibers, including a larger surface area, higher number of active sites, and faster electron transfer rate, the ECL sensor exhibited high sensitivity, a strong linear relationship between peak intensity and the concentration of difenidol hydrochloride, a low detection limit, and good reproducibility and repeatability under optimal conditions. Moreover, the modified electrodes exhibited good performance in terms of recovery. In conclusion, the proposed sensor has wide application prospects for the detection of difenidol hydrochloride.

## Figures and Tables

**Table 1:** Diffusion coefficient of difenidol on different electrodes.

Electrodes	Regression Equation	R^2^	Diffusion Coefficient (cm^2^/s)
Bare GCE	*I* = −4.224 × 10^−8^ + 3.104 × 10^−7^ t^−1/2^	0.993	1.464 × 10^−10^
3D-CNF GCE	*I* = −1.238 × 10^−6^ + 4.053 × 10^−6^ t^−1/2^	0.997	2.496 × 10^−8^

**Table 2:** Comparison of different methods used for the detection of difenidol hydrochloride.

Methods	Linear Range (mol·L^−1^)	Detection Limit (mol·L^−1^)	References
HPLC	3.58 × 10^−5^–1.08 × 10^−4^	—	[5]
LC-MS/MS	2.89 × 10^−9^–2.89 × 10^−7^	1.45 × 10^−9^	[7]
Post-chemiluminescence	1.73 × 10^−8^–2.89 × 10^−7^	5.78 × 10^−9^	[8]
CE-ECL	1.00 × 10^−6^–6.00 × 10^−5^	1.00 × 10^−7^	[29]
ECL	1.00 × 10^−6^–3.30 × 10^−5^	5.00 × 10^−7^	[30]
ECL	1.00 × 10^−7^–1.00 × 10^−4^	6.49 × 10^−10^	[42]
ECL	3.00 × 10^−8^–2.00 × 10^−5^	8.64 × 10^−10^	This work

HPLC: high-performance liquid chromatography, LC-MS/MS: liquid chromatography-mass spectrometry, CE: capillary electrophoresis.

**Table 3:** Detection of difenidol hydrochloride in real samples and their recoveries.

Sample (×10^−6^ mol·L^−1^)	Added (×10^−6^ mol·L^−1^)	Actual (×10^−6^ mol·L^−1^)	Founded (×10^−6^ mol·L^−1^)	Recovery (%)	R.S.D. (%, *n* = 3)
0	-	-	-	-	-
1.5	1.5	0.75	0.74 ± 0.03	99.10	3.91
	3	1.125	1.15 ± 0.02	102.28	1.77
	6	1.875	1.86 ± 0.02	99.51	1.02
	15	4.125	4.12 ± 0.20	101.10	4.41
	30	7.875	7.83 ± 0.20	98.99	1.97

R.S.D.: relative standard deviation.
